# Mycobacterium marinum Infection and Interferon-Gamma Release Assays Cross-Reactivity: A Case Report

**DOI:** 10.7759/cureus.21420

**Published:** 2022-01-19

**Authors:** Premalkumar M Patel, Nicholas Camps, Cynthia I Rivera, Claudio Tuda, Garrett VanOstran

**Affiliations:** 1 Infectious Disease, Mount Sinai Medical Center, Miami Beach, USA; 2 Internal Medicine, Mount Sinai Medical Center, Miami Beach, USA

**Keywords:** ssti, att resistance, igra cross reactivity, mycobacterium marinum, ntm

## Abstract

*Mycobacterium marinum (M. marinum) *is a pathogen that causes skin and soft tissue infections in people who work with contaminated water, such as fish handlers. *M. marinum* infection is mostly limited to patients with compromised immune systems. As per current guidelines, susceptibility testing is not routinely recommended^,^ although sporadic cases have been reported with resistance to routinely prescribed anti-tuberculous drugs. We report a case of a 61-year-old male taking adalimumab with ulcerative skin and soft tissue infection with positive interferon-gamma release assays (IGRA) identified as *M. marinum* and the treatment challenges involved in this case.

## Introduction

*Mycobacterium marinum (M. marinum)* was first isolated in an aquarium in Philadelphia in 1926 from tubercles obtained at necropsy of dead saltwater fish [1,2]. *M. marinum* is a pathogen known to cause skin and soft tissue infections in persons working with contaminated water, such as fish handlers [3]. *M. marinum* infection is mostly limited to patients with compromised immune systems [4]. As per current guidelines, susceptibility testing is not routinely recommended, although sporadic cases have been reported with resistance to routinely prescribed anti-tuberculous drugs [3]. We report a case of a 61-year-old male taking adalimumab with ulcerative skin and soft tissue infection with positive interferon-gamma release assays (IGRA) identified as *M. marinum* and the treatment challenges involved in this case.

## Case presentation

**Figure 1:**
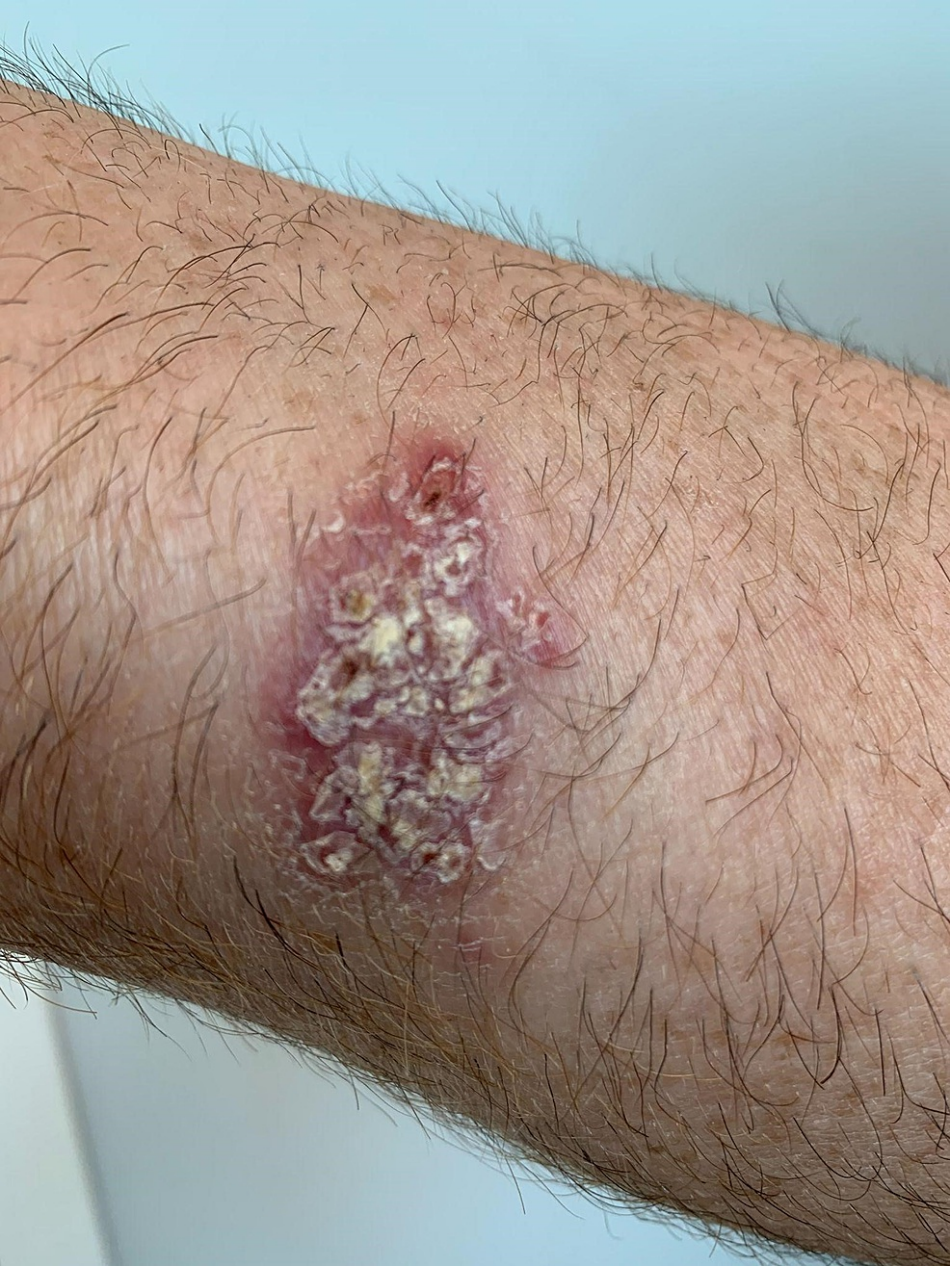
Pre-treatment ulcerative skin lesion due to Mycobacterium marinum infection

**Figure 2:**
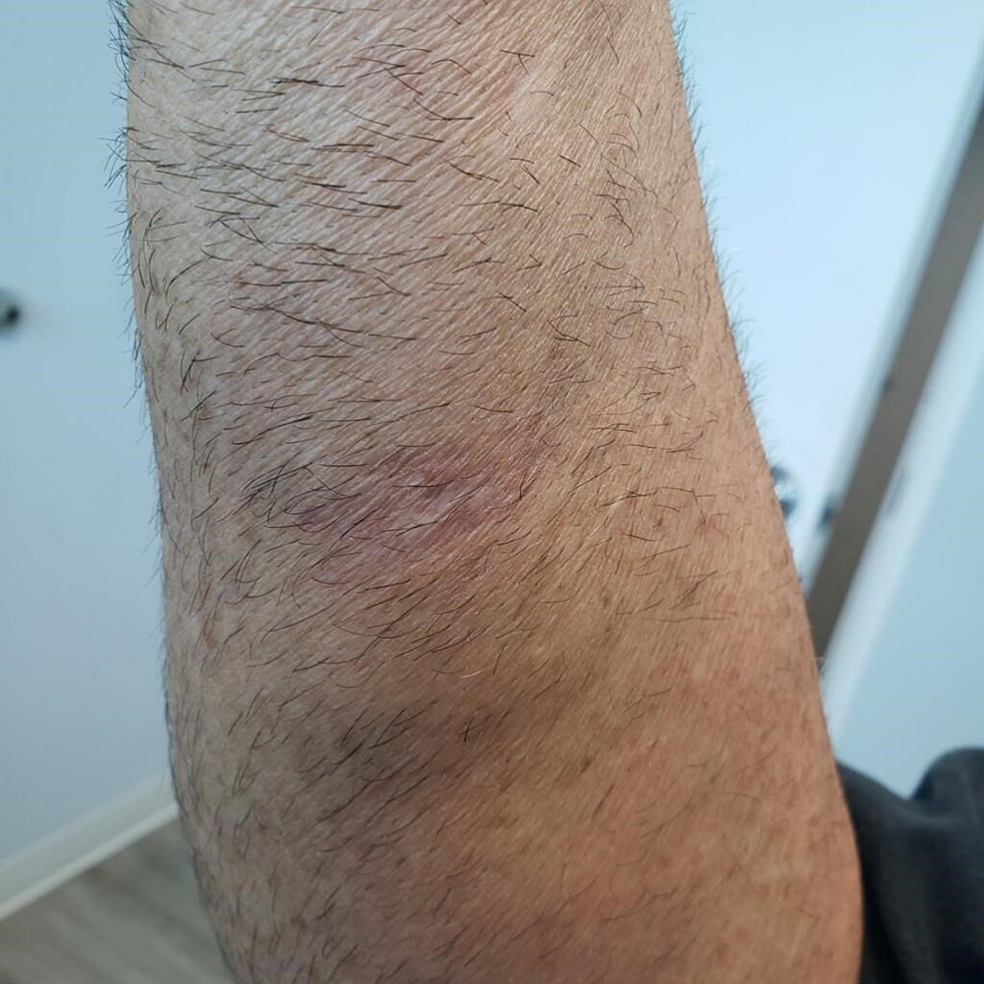
Resolution of skin lesion post susceptibility guided antimicrobial treatment

A 61-year-old male working as a plumber presented with a significant history of psoriasis with psoriatic arthritis on adalimumab taken for three months with prior negative IGRA. His adalimumab was discontinued due to an ulcerative skin lesion. His previous medical history was significant for hyperlipidemia, squamous cell carcinoma, recurrent skin cancer requiring surgical resections was referred to our office for non-tuberculosis mycobacterium (NTM) skin and soft tissue infections (SSTI). The patient had developed a skin lesion atypical to his other recurrent skin lesions on his right distal forearm on the palmar aspect. The lesion was started as a small blister-like lesion which upon removal/resection two months prior became ulcerative (Figures 1, 2). He tried a course of doxycycline for two weeks without improvement. He reports since his resection the lesion has become more erythematous and larger in diameter. He was started empirically on ciprofloxacin and doxycycline for two weeks without much change in the lesion. Repeat IGRA ordered by a dermatologist was positive and initial preliminary culture was acid-fast bacilli (AFB) positive within one week.

The biopsy culture was reported as *M. marinum* and treatment was adjusted to azithromycin and rifampin based on current guidelines. He developed diffuse diarrhea requiring a medication adjustment to ethambutol and rifampin. This was further adjusted to rifabutin and ethambutol based on susceptibility testing (Table 1). He reported blurry vision after a few weeks of treatment. He had a brief interruption in therapy following the initial two to three months due to coronavirus disease 2019 (COVID-19) infection and development of a bacterial nasal abscess. After resuming therapy, he restarted adalimumab due to the progression of his psoriatic arthritis. While on therapy, he had continued resolution of the lesion with less scar formation and clearance of the proximal satellite cold abscess. After completion of five months of treatment, he had resolution of his previously reported ocular symptoms, likely related to an ethambutol drug reaction.

**Table 1:** Mycobacterium marinum susceptibility report S: susceptible; I: intermediate; R: resistant; AFB: acid-fast bacilli; MIC: minimum inhibitory concentrations

Mycobacterium ID and susceptibility			
Source: proximal forearm			
AFB identification: *Mycobacterium marinum* (identified by DNA sequencing)			
Antibiotics	MIC	Susceptibility	Unit
Amikacin	4	S	mcg/mL
Ciprofloxacin	4	R	mcg/mL
Clarithromycin	2	S	mcg/mL
Doxycycline	8	R	mcg/mL
Ethambutol	2	S	mcg/mL
Ethionamide	1.2	I	mcg/mL
Isoniazid	>8	I	mcg/mL
Linezolid	4	I	mcg/mL
Moxifloxacin	2	S	mcg/mL
Rifampin	2	R	mcg/mL
Rifabutin	≤0.25	S	mcg/mL
Streptomycin	16	I	mcg/mL
Trimeth/sulfa	>8/152	R	mcg/mL

## Discussion

The incidence of *M. marinum* skin infections is increasing. This organism is a pathogen causing tuberculosis-like illness in fish, infecting humans through exposure by injured skin specifically in persons working in a contaminated aqueous environment [5,6]. The incidence of skin infections caused by *M. marinum* is increasing dramatically, mainly due to the increasing prevalence of HIV infections and the use of immunosuppressive therapies. The use of tumor necrosis factor inhibitors, for example, is associated with an increased risk of tuberculosis and of infection caused by NTM [7]. Additional defects in T-helper cell or macrophage function may predispose patients to NTM disease.

Interferon-gamma releasing assay is a test used widely for screening tuberculosis. It measures the release of gamma interferon by white blood cells. The limitation of the IGRA as a test is the inability to differentiate between latent tuberculosis infection and active tuberculosis. The QuantiFERON-TB-Gold test is based on response to the *Mycobacterium tuberculosis* (MTB)-specific peptide antigens ESAT-6, CFP-10, and TB7.7, which are located in a specific genomic area in MTB, called the region of difference (RD1). The RD1 is present in mycobacteria belonging to the *Mycobacterium tuberculosis* complex (*M. tuberculosis*, *Mycobacterium bovis*, *Mycobacterium canettii*, *Mycobacterium africanum*, *Mycobacterium pinnipedii*, *Mycobacterium caprae*, *Mycobacterium microti*, *Mycobacterium mungi*, and *Mycobacterium orygis*) and very few NTM species also share the RD1 of MTB (*M. marinum*, *Mycobacterium gastri*, *Mycobacterium kansasii*, *Mycobacterium riyadhense*, and *Mycobacterium szulgai*). Infection with these strains can potentially result in a positive QuantiFERON-TB Gold test (QFT) result [8]. The virulence determinants of the *M. marinum* are cell wall-associated lipid phthiocerol dimycocerosates, phenolic glycolipids, and ESAT-6 secretion system 1 (ESX-1) [9]. We feel strongly that our patient likely had a cross-reactive IGRA response due to *M. marinum* SSTI given his prior negative result before starting adalimumab and subsequent positivity with infection.

As per the American Thoracic Society/Infectious Disease Society of America (ATS/IDSA) guideline, initial susceptibility testing is not required for *M. marinum* skin infections. Susceptibility testing is recommended if initial treatment fails. Though resistance potential is very low to standard anti-tuberculosis medications, some resistant cases have been reported earlier. As evidenced in our case, initial empiric treatment options based on 2007 guidelines revealed multiple resistances to sulfonamide, tetracycline, and even rifampin [3]. In patients with immunocompromised conditions, a combination of therapy is required for several months depending on the site and severity of infection [10]. Compliance monitoring, drug interaction monitoring, and managing potential drug reactions are a few but challenging tasks in management.

## Conclusions

Our case illustrates effective second-line therapy based on susceptibilities and can serve as a model for future *M. marinum* SSTI cases. We assert that until guidelines are updated, susceptibility testing should be obtained to guide therapy, especially in severe infections. Routine screening with IGRA in nonendemic areas is problematic. Strong consideration for culture analysis should be stressed as the gold standard for the diagnosis and speciation of NTM organisms. As discussed previously, the cross-reactivity potential with IGRA and aforementioned NTM organisms is frequently overlooked. This could lead to additional testing for MTB in patients presenting with atypical NTM infections. We hope this case serves to highlight the importance of knowing cross-reactivity to certain NTM pathogens exists clinically.
